# 378. Descriptive Analysis of SARS-CoV-2 Infections Among Health System and University Employees

**DOI:** 10.1093/ofid/ofab466.579

**Published:** 2021-12-04

**Authors:** Jessica Seidelman, Ibukunoluwa Akinboyo, Maya Rinehart, Rebekah W Moehring, Deverick J Anderson, Kristen Said, Carol A Epling, Sarah S Lewis, Becky Smith, Matthew Stiegel

**Affiliations:** 1 Duke University, Durham, NC; 2 Duke University Health System, Durham, NC; 3 Duke Center for Antimicrobial Stewardship and Infection Prevention, Durham, NC; 4 Duke University Medical Center, Durham, NC

## Abstract

**Background:**

We aimed to describe SARS-CoV-2 (COVID-19) infections among employees in a large, academic institution.

Table 1. COVID-19 Attribution Definitions

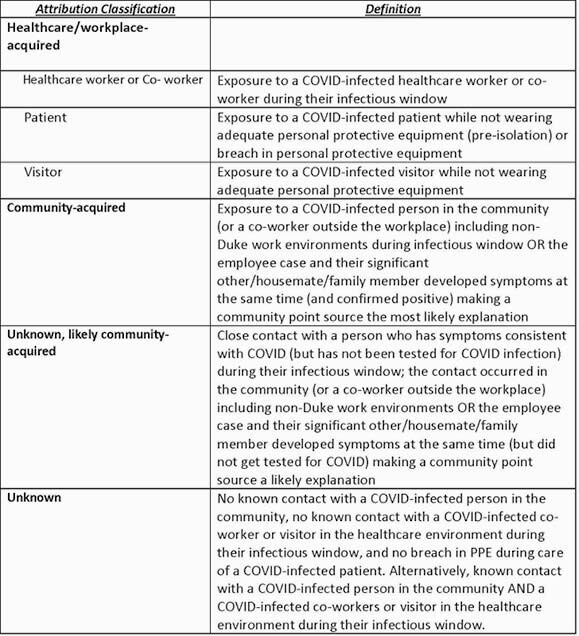

Table 2. Description of 3,140 COVID 19 Infections in Employees from 3/2020 to 4/2021

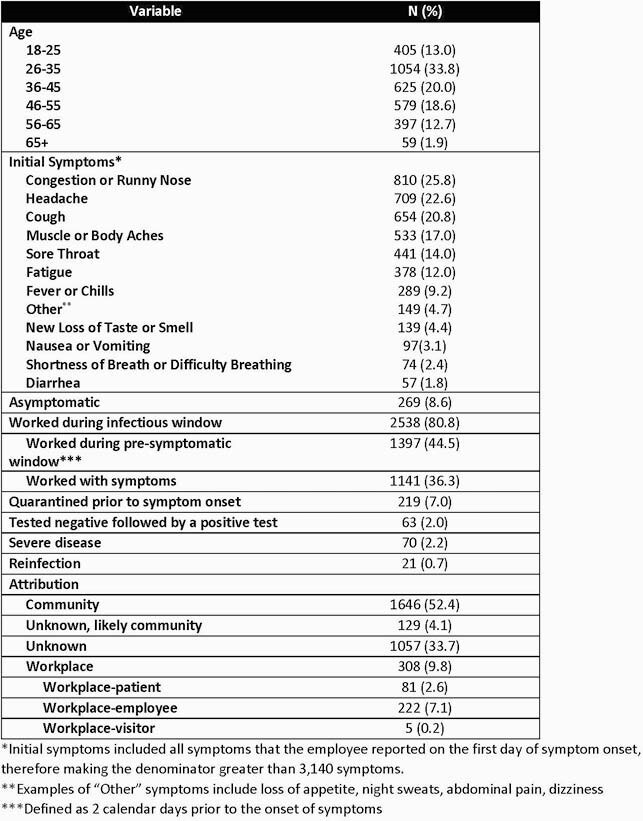

**Methods:**

We prospectively tracked and traced COVID-19 infections among employees across our health system and university. Each employee with a confirmed positive test and 3 presumed positive cases were interviewed with a standard contact tracing template that included descriptive variables such as high-risk behaviors and contacts, dates worked while infectious, and initial symptoms. Using this information, the most likely location of infection acquisition was adjudicated (Table 1). We compared behavior frequency between community and unknown, likely community and community and unknown cases using descriptive statistics.

Table 3. Risk Factors for Community, Likely Community, and Unknown Cases

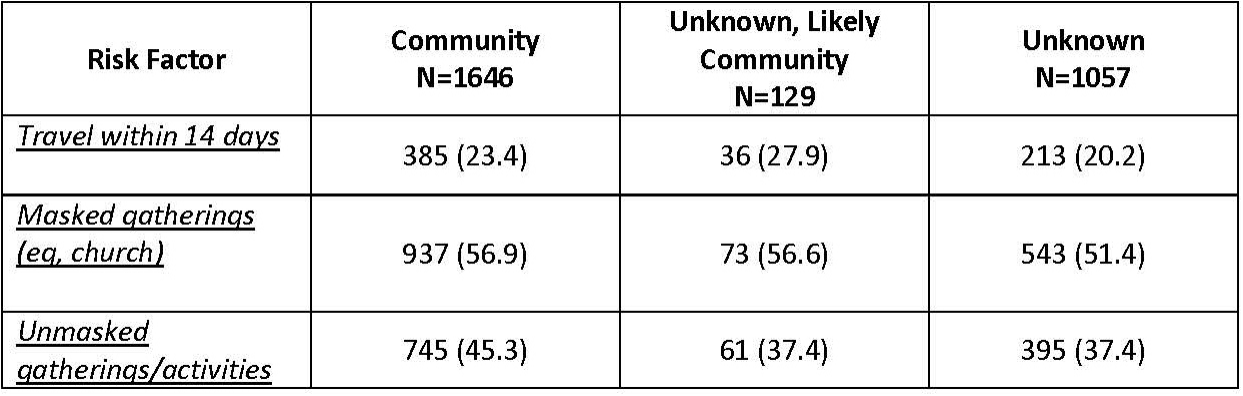

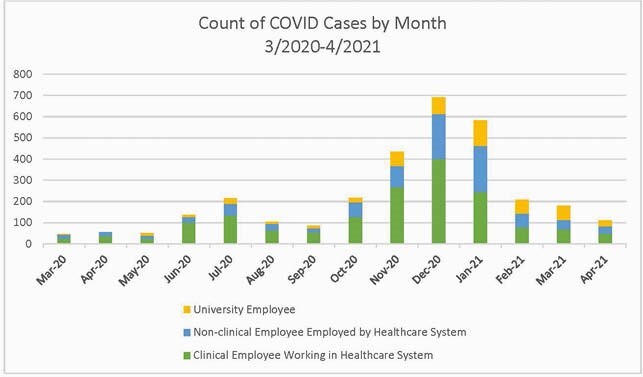

Number of SARS-CoV-2 cases among employees between 3/2020 and 4/2021 by month and stratified according to clinical employee working in the healthcare system, non-clinical employee employed by the healthcare system, and university employee

**Results:**

From 3/2020 to 4/2021 we identified 3,140 COVID-19 infections in 3,119 employees out of a total of 34,562 employees (9.0%) (Figure 1). Of those 3,119 employees 1,685 (54.0%) were clinical employees working in the health system, 916 (29.4%) were non-clinical employees working in the health system, and 518 (16.6%) were university employees. Descriptive characteristics for the COVID-19 infections and adjudications are outlined in Table 2. Severe disease among employees was significantly less frequent compared to patients in the health system (15.3% vs 2.2%, p< 0.01). The frequency of travel within 14 days, masked gatherings and unmasked gatherings/activities was not significantly different between the community and unknown, likely community groups or the community and unknown groups (Table 3).

**Conclusion:**

The majority of COVID-19 infections were linked to acquisition in the community, and few were attributed to workplace exposures. Employees with unknown sources of COVID-19 participated in higher-risk activities at approximately the same frequency as employees with community sources of COVID-19. The most frequently reported initial symptoms were mild and non-specific and rarely included fever. Despite a comprehensive testing and benefit program, a large proportion of COVID-positive employees worked with symptoms, highlighting ongoing challenges with presenteeism in healthcare.

**Disclosures:**

**Rebekah W. Moehring, MD, MPH**, **UpToDate, Inc.** (Other Financial or Material Support, Author Royalties)

